# Toward smart design of retinal drug carriers: a novel bovine retinal explant model to study the barrier role of the vitreoretinal interface

**DOI:** 10.1080/10717544.2017.1375578

**Published:** 2017-09-19

**Authors:** Karen Peynshaert, Joke Devoldere, Valérie Forster, Serge Picaud, Christian Vanhove, Stefaan C. De Smedt, Katrien Remaut

**Affiliations:** a Lab of General Biochemistry and Physical Pharmacy, Faculty of Pharmaceutical Sciences, Ghent University, Ghent, Belgium;; b Ghent Research Group on Nanomedicines, Ghent University, Ghent, Belgium;; c Institut de la Vision, INSERM, Université Paris 6, Paris, France;; d Department of Respiratory Medicine, Ghent University, Ghent, Belgium

**Keywords:** Vitreoretinal interface, retinal gene delivery, inner limiting membrane, vitreous, retinal explant, *ex vivo*, large animal, nanoparticle

## Abstract

Retinal gene delivery via intravitreal injection is hampered by various physiological barriers present in the eye of which the vitreoretinal (VR) interface represents the most serious hurdle. In this study, we present a retinal explant model especially designed to study the role of this interface as a barrier for the penetration of vectors into the retina. In contrast to all existing explant models, the developed model is bovine-derived and more importantly, keeps the vitreous attached to the retina at all times to guarantee an intact VR interface. After *ex vivo* intravitreal injection into the living retinal explant, the route of fluorescent carriers across the VR interface can be tracked. By applying two different imaging methods on this model, we discovered that the transfer through the VR barrier is size-dependent since 40 nm polystyrene particles are more easily taken up in the retina than 100 and 200 nm sized particles. In addition, we found that removing the vitreous, as commonly done for culture of conventional explants, leads to an overestimation of particle uptake, and conclude that the ultimate barrier to overcome for retinal uptake is undoubtedly the inner limiting membrane. Damaging this matrix resulted in a massive increase in particle transfer into the retina. In conclusion, we have developed a highly relevant *ex vivo* model that maximally mimics the human *in vivo* physiology which can be applied as a representative test set-up to assess the potential of promising drug delivery carriers to cross the VR interface.

## 1. Introduction

Today, blindness affects 180 million people worldwide. Next to the tragic impact of vision loss on the lives and the surroundings of these people, the health care costs associated with blindness are immense (International AMD Alliance, 2010). Since many blinding pathologies find their origin in the retina, a vast amount of research in academics and industry is dedicated to the delivery of therapeutic entities (e.g. steroids, antibodies, or nucleic acids) to the back of the eye. For mutation-dependent pathologies, gene therapy has made a great leap forward over the last decade. As leading example, treatment of RPE65-dependent Leber congenital amaurosis (LCA) by subretinal injection of adeno-associated viral vectors (AAV) has shown promising results in Phase 3 clinical trials and is expected to reach the clinic soon (Schimmer & Breazzano, 2015; Spark Therapeutics Press Release, 2016). However, subretinal injections are mainly efficient to reach cells surrounding the injection spot, i.e. photoreceptors or RPE cells. Hence, the majority of retinal gene therapy trials are focused on treatment of the outer retina. Nevertheless, the inner retina harbors important target cells as well including retinal ganglion cells (RGCs) as target for Leber Hereditary Optic Neuropathy (LHON) (Boye et al., 2013), Müller cells for neurotrophic strategies (Gauthier et al., 2005), and bipolar and amacrine cells for optogenetic therapy (Busskamp et al., 2012). It is, however, not at all evident to reach these cell types via subretinal injection.

**Figure 1.**
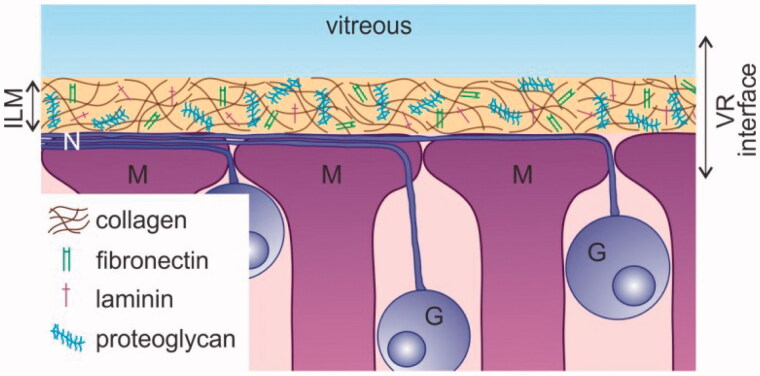
Schematic drawing of the vitreoretinal interface. G: Ganglion cell; ILM: inner limiting membrane; M: Müller cell; N: nerve fiber; VR: vitreoretinal.

An alternative method to reach the inner retina is intravitreal (IVT) injection, a technique that is considered safe, minimally invasive and relatively easy to perform (Englander et al., 2013). Nonetheless, the transfer of (high molecular weight) drugs and nanosized particles carrying drugs into the retina after IVT injection remains troublesome, primarily because of the presence of the vitreoretinal (VR) interface. As shown in Figure 1, this interface consists of three structures: peripheral vitreous, the inner limiting membrane (ILM), and Müller cell endfeet. The vitreous, a transparent gel composed of collagen fibrils filled up with hyaluronic acid, may hamper the mobility of carriers preventing them to reach the retina (Peeters et al., 2005; Le Goff & Bishop, 2008; Da Costa et al., 2016). The ILM has an extracellular matrix structure composed of a collagen IV network intertwined with proteoglycans, laminin, and fibronectin (Halfter et al., 2008; Bu et al., 2015). It represents a physical border between the vitreous and the retina and functions as a sieve that more than often impedes the transfer of drug carriers into the retina (Pitkänen et al., 2004; Koo et al., 2012; Gan et al., 2013). Once drug carriers pass the vitreous and ILM they encounter retinal cells, where the first cell type they face is likely the Müller cell, a glial cell of which the endfeet align with the ILM. If these retinal cells efficiently internalize the drug carriers, retinal delivery can finally be regarded as successful.

Aiming for the delivery of nucleic acids in retinal cells, several groups have reported successful penetration of both non-viral as well as viral gene carriers through the entire VR interface of rodents, resulting in gene expression in the retina. In animals larger than rodents, however, IVT injection rarely results in effective gene expression (Pitkänen et al., 2004; Hellström et al., 2009; Koo et al., 2012; Mowat et al., 2014; Boyd et al., 2016; Takahashi et al., 2017). Interspecies differences greatly hamper the extrapolation of successful IVT therapies in rodents to larger animal models and humans. This is likely because the VR interface is substantially more difficult to overcome in larger species (Trapani et al., 2015). Indeed, the anatomy of the rodent eye and the structure of its vitreous and ILM is less representative for human physiology (Xu et al., 2013; The Lasker/IRRF Initiative for Innovation in Vision Science, 2014). As an example, the thickness of the mouse ILM is estimated to be around 100 nm, while a parafoveal thickness of up to 4 µm is measured in human eyes (Henrich et al., 2012; Halfter et al., 2015). In light of this, some research groups perform drug delivery studies directly on explants of larger species (Kobuch et al., 2008; Fradot et al., 2011). Nevertheless, in these studies the vitreous is separated from the retina so that information regarding the VR interface is lost. Therefore, there is an urgent need for models with an intact VR interface based on larger animals since it is expected these will be more representative for the human VR interface. Such models will become highly useful in research which aims for drug delivery into the retina.

To address this need, we developed an *ex vivo* explant model that is bovine-derived and most importantly, guarantees an intact VR interface by keeping the vitreous attached to the retina at all times. Drugs or drug carriers can be injected *ex vivo* into the vitreous of the VR explant after which their potential to cross the VR interface as well as their transport route into the retina can be examined by microscopy. In this paper, we present the methodology of this novel VR explant and validate its retinal morphology and viability. Furthermore, we demonstrate the potential of the model in drug delivery research by studying how the size of nanosized polystyrene beads (as a model for nanoparticles carrying drugs) influences their transport over the VR interface. Finally, we apply the presented model to draw conclusions on the drug delivery barrier role of the separate parts of the VR interface.

## 2. Methods

### 2.1. Materials

Carboxylated polystyrene beads (FluoSpheres^®^) were purchased from Molecular Probes™: 40 nm (8795), 100 nm (F8800), 200 nm (F8809). Dyes for Müller cell and viability staining were obtained from Invitrogen: Hoechst 33342 (H3570), FM^®^ 1-43 (T3163), Mitotracker^®^ Deep Red (M22426), Propidium iodide (P3566). Antibodies against glutamine synthetase (ab73593) and Collagen IV (ab6586) were purchased from Abcam; AlexaFluor^®^ 647 tagged secondary antibody (A27040) was obtained from Invitrogen. Cell culture materials were mostly acquired from Gibco™: CO_2_ Independent medium (18045088), Neurobasal^®^-A medium (10888022), Advanced DMEM medium (12491023), B-27^®^ supplement (17504044), Penicillin–streptomycin (15140122), l-Glutamine (25030081), Trypsin–EDTA 0.25% (25200072).

### 2.2. Nanoparticle characterization

The hydrodynamic size and zeta potential of the FluoSpheres^®^ are determined using a Malvern Zetasizer Nano (Malvern Instruments, Worcestershire, U.K.). For this purpose, the FluoSpheres^®^ are diluted a thousand times in HEPES buffer (25 mM, pH 7.2) prior to performing the measurements at 25 °C. Size measurements are done in triplicate with three runs per replicate, and presented based on the number distribution. The zeta potentials are calculated from the electrophoretic mobility of the FluoSpheres based on the Henry equation considering the Smoluchowski approximation. Zeta potential measurements are done in triplicate with two runs per replicate.

### 2.3. Dissection and culture of a *conventional* retinal explant

Fresh bovine eyes are obtained from the local slaughterhouse where they are enucleated up to 15 min after the animal is sacrificed. The eyes are transported and kept in ice cold CO_2_ independent medium until dissection. After removing all extra-ocular connective tissue and disinfecting the eyes by soaking them in 20% ethanol, the sclera is punctured with a 21G needle around 10 mm below the limbus. This hole next serves as an entry point for the scissors used to bisect the eye. After bisection the eye, the vitreous is removed and the posterior eye cup is filled with cold CO_2_ independent medium. Next, 3–4 flaps are cut in the eyecup, preferably along large veins. While the whole structure is submerged in medium, a trephine blade (Beaver^®^) with 10 mm diameter is used to isolate a circular piece of retina from each flap. The explant is then removed from the eyecup by gently pipetting medium below. Two of these explants are then placed on a dry 75 mm Transwell^®^ explant filter after which the explant filter is moistened with explant culture medium (Neurobasal^®^-A, 1% B-27^®^ supplement, 1% Penicillin–streptomycin, 0.5% l-glutamine) and 10 ml of the same medium is added below the explant filter. Finally, the explants are incubated at 37 °C and 5% CO_2_.

### 2.4. Dissection and culture of a *vitreoretinal* explant

**Figure 2.**
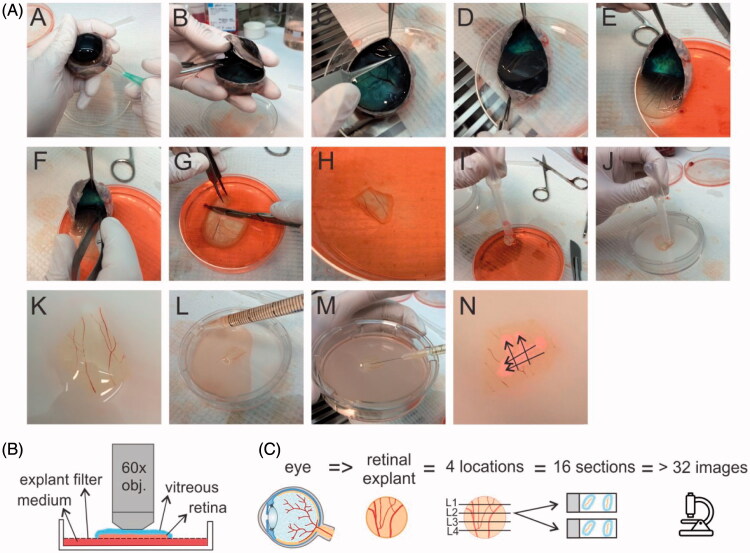
(A) Step-by-step overview of the dissection protocol to culture a bovine vitreoretinal explant. (B) set-up for ‘direct imaging’ of the vitreoretinal explant. (C) Workflow applied for cryosectioning of the retina and cryosection imaging.

Our newly developed dissection protocol differs from the conventional one by the preservation of vitreous and an intact ILM during dissection. The preparation of this so-called ‘vitreoretinal explant’ is shown in Figure 2(A). Before dissection, isolated bovine eyes are gently warmed by 20–30 minutes incubation in CO_2_ independent medium at room temperature, a crucial step to allow smooth separation of the retina from the RPE-choroid layer in step E of the protocol. As for conventional explants, the sclera is punctured with a 21G needle around 10 mm below the limbus (step A). In step b, the eye is bisected so that a posterior eye cup filled with vitreous gel remains. Next, at the rim of the eyecup, the retina is gently detached from the choroid using a fine pincet, except at the side of the optic nerve (step C). Then, the vitreous is gently pulled down during which the attached retina slides along (step D–E). After cutting the optic nerve (step f), the whole tissue is slid with vitreous side upwards into a culture dish of 10 cm (Corning) filled with cold CO_2_ independent medium. In step g, a scalpel is used to cut up to three pieces of VR explant (up to 1.5 cm^2^) from the vitreous side downwards through the retina. Hereby the potentially damaged edges of the retinal tissue are avoided. In step I–K, a plastic Pasteur pipette with a cut tip is used to gently aspire the VR explant and to transfer it to a dry explant filter from a 75 mm Transwell^®^ dish (3419, Corning). Excess amounts of vitreous (e.g. lying next to the retina) can then be removed by aspiration of some vitreous using a plastic Pasteur pipette while cutting through the gel with scissors. Finally, 10 ml of supplemented Neurobasal^®^-A medium is added below the explant filter (Step L), and the VR explant is placed in an incubator with 5% CO_2_ at 37 °C.

### 2.5. *Ex vivo* injection of nanomaterials

Immediately after explant dissection, carboxylated PS beads (1.42*10^15^ nanoparticles/ml) were injected (using a 30G needle) in the vitreous layer covering the retina. Multiple injections (about 50 µL per injection) within one VR explant were performed. The injections were done ‘horizontally’ to avoid direct transfer of nanoparticles into the retina (Figure 2(A), M). As shown in step N, each intravitreal injection typically resulted in an ‘injection band’ (arrows) containing a high concentration of (fluorescent) beads. Following injection, the beads then diffuse through the vitreous toward the retina. Since IVT injection is not possible in conventional retinal explants (as there is no remaining vitreous), 25 µl of the carboxylated PS beads was applied on top of the explants. All explants were incubated with the PS beads at 37 °C for 24 hours.

### 2.6. Explant staining, fixation and ‘direct imaging’

Explants were stained by the injection of a mixture of dyes in the vitreous covering the retina, followed by an incubation at 37 °C for 2 hours. Nuclei were stained with 10 µg/ml Hoechst, lipid membranes with 20 µg/ml FM-43 and Müller cells with 2 µM Mitotracker Deep red following the protocol of Uckermann et al. (2004). To evaluate retinal cell viability, a mixture of Propidium Iodide and Hoechst was added to the explant culture medium below the explant filter resulting in a final concentration of 10 µg/ml for both dyes. After dye incubation, the explants were fixed by replacing the explant culture medium with 4% paraformaldehyde (in PBS) during 2 hours at 4 °C. After fixation, the fixative was replaced by PBS for imaging.

Fixed *ex vivo* explants were imaged with a confocal microscope (C1-si, Nikon) directly or after preparing cryosections (see below). For ‘direct imaging’, a 60× water dipping objective (NIR, Apo) with a large working distance (2.8 mm) was pushed on top of the vitreous which allowed to image from the vitreous until the ganglion cell layer (Figure 2(B)).

### 2.7. Explant cryosectioning and immunohistochemistry

After fixation, the fixative below the explant filter is removed and replaced by 30% sucrose and incubated overnight at 4 °C. After snap freezing the samples in Tissue-Tek^®^ O.C.T. (Sakura) with liquid nitrogen, 14 µm sections were cut with a cryostat (Leica). For reliability of our results, we applied the workflow drawn in Figure 2(C): 16 sections were analyzed per retina, resulting from 4 distinct retina locations. These retinal sections were permeabilized with 0.1% Triton for 5 min prior to a 1 hour incubation at room temperature with 5% goat serum in PBS as a blocking step. Next, sections were incubated overnight at 4 °C with 1:200 rabbit antibody against Collagen IV. Finally, after a 1 hour incubation at room temperature with goat anti-rabbit Alexafluor 647 conjugated secondary antibody and 10 µg/ml Hoechst the sections were mounted with Vectashield (Vector Laboratories) and prepared for imaging. The ‘cryosection imaging’ was done with a confocal microscope (C1-si, Nikon) using a 10× objective (CFI Plan Apochromat, Nikon) and a 60× water objective (NIR Apo, Nikon).

### 2.8. Uptake of nanoparticles by the retina: cryosection image processing for semi-quantitative analysis

To analyze the uptake of the PS beads by the retina, following the workflow presented in Figure 2(C), (minimally) two images were taken per retinal section, resulting in at least 32 images per retinal explant. Image analysis was done with FIJI (NIH) as follows: using the Polygon Tool, a region of interest (ROI) was drawn around the retinal area below the ILM. Then, the area outside this ROI, i.e. above the ILM, was colored black using the ‘clear tool’ so only particles present within the ROI (retina) would be counted. The number of particles within the ROI was counted using the ‘analyze particles’ tool. Finally, the surface area of the ROI was measured by applying the ‘measure’ tool in the ROI manager so that the number of counted particles could be recalculated per 1000 µm^2^. Based on these results, the various sections were categorized in three categories i.e. samples containing a low (<10), higher (10–30) or very high (>30) amount of particles per 1000 µm^2^.

## 3. Results

### 3.1. Vitreoretinal explant morphology and viability

**Figure 3.**
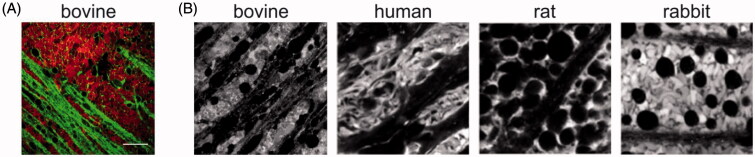
(A) Confocal view on the GCL/NFL layer as obtained through ‘direct imaging’. Müller cells (red) are stained with Mitotracker Deep Red, lipid membranes and nerve fibers (green) with FM-43. Scale bar: 20 µm. (B) Confocal view onto the GCL/NFL layer of different species stained for Müller cells. Bovine retinal tissue was stained by Mitotracker Deep Red (own data); examples of human, rat and rabbit tissue were stained by Mitotracker Orange and taken from (Uckermann et al., 2004).

VR explant differs from the conventional explant by the presence of an intact VR interface (e.g. vitreous and ILM). Despite this seemingly small difference, the search for a dissection protocol suitable to obtain the VR explant was intensive. The dissection protocol as presented in Figure 2(A) resulted in an explant that keeps a layer of vitreous attached to the retina. Next, we visualized the ganglion cell layer (GCL) and nerve fiber layer (NFL) of the VR explant by staining for Müller cells and lipid membranes. Figure 3(A) shows that a large portion of this layer is taken in by a patchwork of Müller cell endfeet (red color) separated by lipid membranes (green color). Nerve fibers run unidirectional through these patchworks, while other dye-less cell types such as RGCs are randomly scattered within this layer.

When we compare the morphology of the bovine GCL/NFL layer with the morphology of Mitotracker-stained tissue of others species as presented in literature (Figure 3(B)), the morphology of the bovine GCL/NFL layer highly relates to that of the human retina. Interestingly, also bovine vitreous has a similar structure as human vitreous and has therefore been frequently applied in *ex vivo* models developed for IVT drug delivery studies (Xu et al., 2000, 2013; Peeters et al., 2005; Martens et al., 2013; Käsdorf et al., 2015). Therefore, both the presence of vitreous and the representative GCL/NFL layer in the bovine-derived VR explant is highly likely of relevance for drug delivery studies in human.

**Figure 4.**
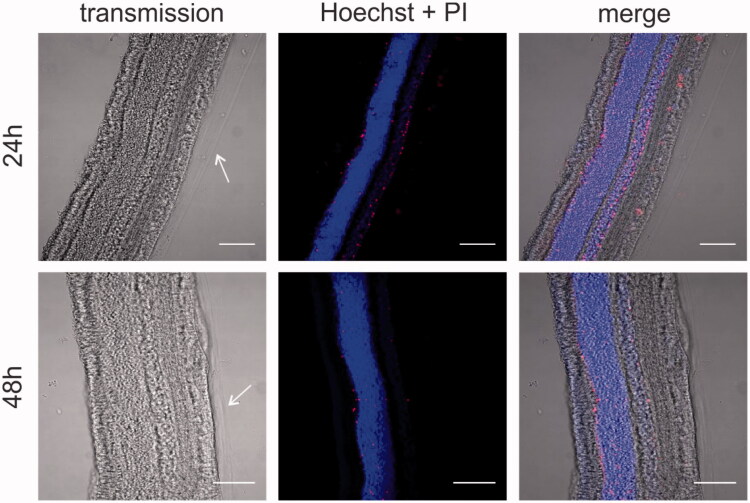
Cryosection imaging showing gross morphology and tissue viability of vitreoretinal explants. Nuclei are stained with Hoechst (blue), dead cells are stained with propidium iodide (red). Arrows indicate the vitreous layer. Scale bar: 100 µm.

Next, we aimed to assess if the dissection protocol and explant culture conditions maintain the integrity and viability of the various retinal layers. VR explants were stained with the nuclear label Hoechst to identify the various layers and check gross retinal morphology. Also, the cell-impermeable (red) dye Propidium Iodide (PI) was added to identify cell viability. The cryosections in Figure 4 show that the architecture of the retina is nicely preserved and all retinal layers can be easily distinguished. In addition, a layer of vitreous gel is clearly attached at the side of the ILM as indicated by the arrows. Also note that the number of PI-positive cells is very limited at both time points from which we conclude that the explant is viable for at least 48 hours.

### 3.2. Influence of nanoparticle size on the transfer through the vitreoretinal interface studied by direct imaging of the VR explant

Following the successful characterization of the morphology and viability of the VR explant, we applied the model to study, for the first time, the size-dependent penetration of polystyrene (PS) beads over the intact VR interface of a large animal. The three selected sizes of the PS beads correlate with the sizes of (intravitreally injected) gene carriers currently under investigation for the treatment of retinal diseases, while 40 nm ranges at the upper size limit of viral vectors, the 100 and 200 nm sized beads represent the size of most common non-viral vectors. Table 1 represents the size and zeta-potential (a measure for the surface charge) of the carboxylated PS beads dispersed in HEPES buffer, as measured by DLS. As expected, the particle sizes were highly reproducible and all particles were negatively charged due to the carboxylated surface. This negative charge is of importance since negatively charged entities are known to diffuse well through the vitreous, increasing their potential to reach the retina.

**Table 1.** Size and zeta-potential of the carboxylated PS beads in HEPES buffer.

	Size (nm)	Zeta potential (mV)
40 nm	37 (±0.4)	−14 (±0.4)
100 nm	90 (±0.4)	−30 (±0.8)
200 nm	205 (±1.2)	−39 (±0.4)

**Figure 5.**
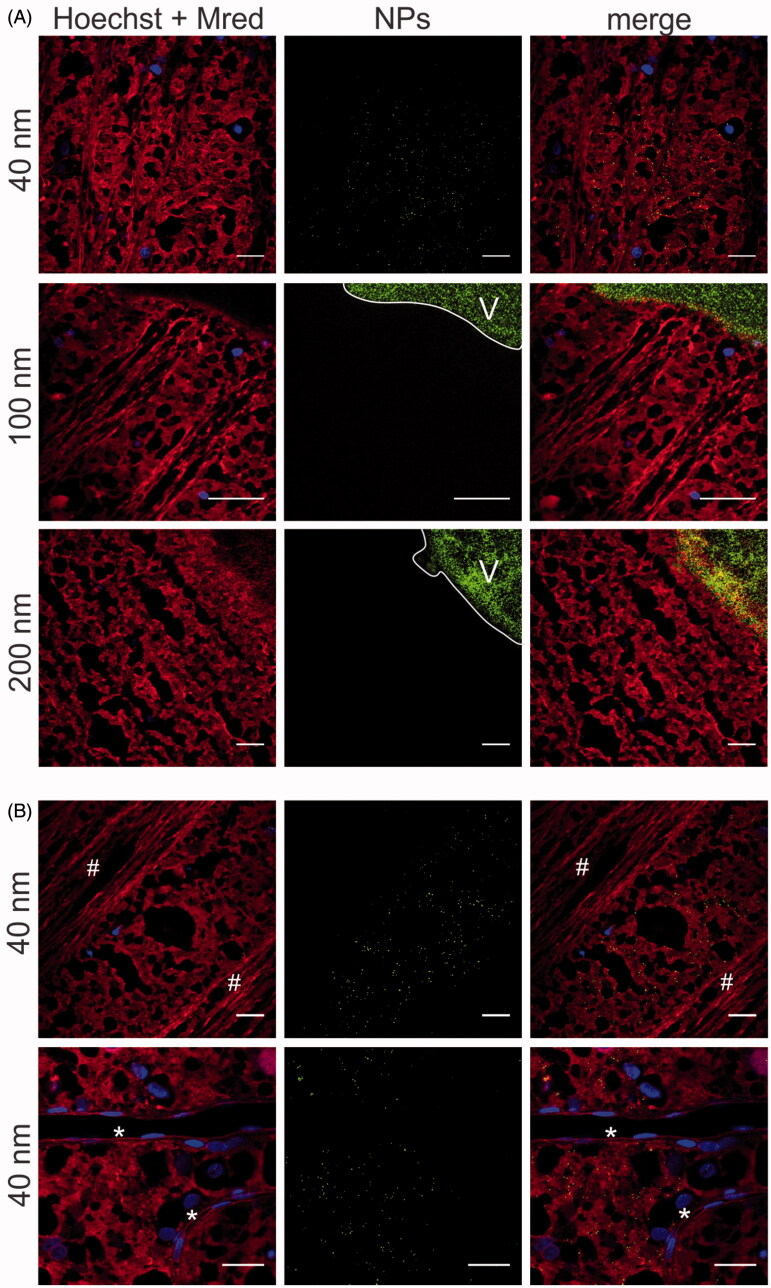
Transfer of (green) PS beads through the VR interface, as visualized by ‘direct imaging’ (Figure 2B). Müller cells (red) are stained with Mitotracker Deep Red, cell nuclei (blue) with Hoechst. (A) 40 nm particles enter the Müller cells, 100 and 200 nm particles remain in the vitreous (indicated with ‘V’). Scale bar: 25 µm. For optimal contrast we refer the reader to the online version. (B) Co-localization of 40 nm PS beads (green) with Müller cells. Asterisks (*) indicate blood vessels, number signs (#) indicate nerve fibers. Scale bar: 25 µm.

As described above, the PS beads were administered into the VR explant by multiple 50 µl *ex vivo* IVT injections. 24 Hours post-injection, the transfer of the NPs from the vitreous into the retina was investigated using the direct imaging method. Here, a water dipping objective is gently pushed on top of the vitreous to image the vitreous and NFL/GCL layer (Figure 2(A)). Figure 5(A) shows the transfer of green PS beads through the VR interface into the NFL/GCL layer (of which the Müller cell endfeet are stained in red).

Our data show that only 40 nm particles can be spotted in the retina, while 100 nm and 200 nm NPs do not appear in the NFL/GCL layer. Note that, on the micro-scale, the position of the retina as it occurs in Figure 3 is not entirely flat. Therefore, often images were obtained in which the vitreous (right top side) appears bright green as it is heavily loaded with green PS beads, while the rest of the image displays Müller cell endfeet and nerve fibers. Remarkably, 40 nm particles that were able of crossing the VR interface tended to selectively co-localize with the Mitotracker-stained Müller cells. Indeed, 40 nm PS beads were only present in the mosaic of red islands representing Müller cells, though not in the blood vessels, nerve fibers or dye-less cells representing other retinal cell types (Figure 5(B)).

### 3.3. Influence of nanoparticle size on the transfer through the vitreoretinal interface studied by cryosection imaging of the VR explant

**Figure 6.**
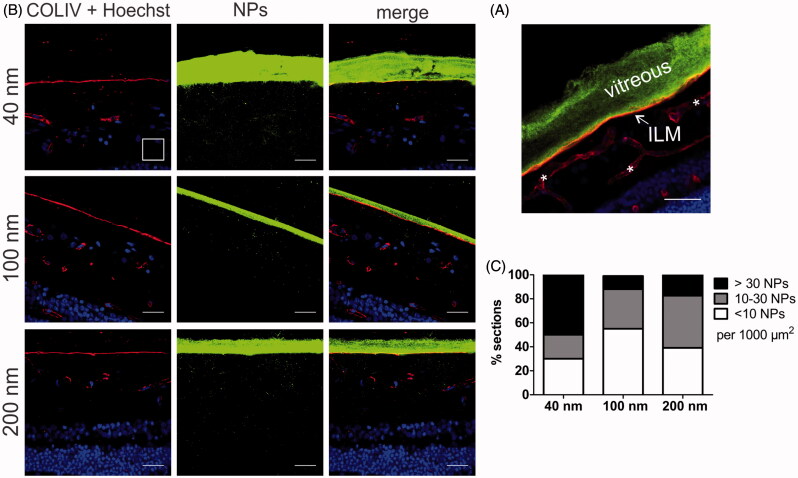
(A) Retinal cross-section of bovine retina with vitreous attached. Green: 100 nm polystyrene beads injected in the vitreous; Red: ILM stained for collagen, which also stains retinal blood vessels (*); Blue: Hoechst staining of nuclei. Scale bar 50 µm. (B) Representative cryosection images showing the transport of PS beads through the vitreoretinal interface, 24 h after injecting the PS beads in the vitreous of the VR explant. ILM and blood vessels are stained by anti-collagen antibodies (red), nuclei (blue) with Hoechst, particles are shown in green. Note that the contrast in the middle panel is enhanced to optimally visualize the PS beads, while the brightness of the PS beads is reduced in the right panel to illustrate the perfect alignment of the vitreous and ILM. For optimal contrast we refer the reader to the online version. The scale square in the top left image represents 1000 µm^2^ (31.6 µm × 31.6 µm). Scale bar: 31.6 µm. (C) Semi-quantitative analysis of transport of PS beads through the VR interface after 24 h incubation. (*n* = 3).

We further investigated the uptake of differently sized PS beads in the VR explant by cryosection imaging (Figure 2(C)). Considering our interest in the VR interface, we visualized the ILM by staining it with (red) antibodies against Collagen IV. Typically this resulted in images as presented in Figure 6(B), where the vitreous, which appears as bright green due to the high number of PS beads, nicely aligns with the intact ILM. Based on these images, we estimated the thickness of the ILM to be around 2 µm, which correlates well with the average thickness of fixed human ILM (∼2 µm) reported before (Halfter et al., 2015).

To rule out any bias, cryosection preparation was performed complying with the workflow drawn in Figure 2(C) followed by image processing, resulting in at least 32 images that were analyzed per given NP size. We opted for this systematic and objective approach based on the initial observation that NP uptake often greatly varied between different explants and even between different locations within one explant. Figure 6(A) shows the number of NPs that penetrated in the retina, subdivided in categories of low (<10 NPs), intermediate (10–30 NPs), and high (>30 NPs) penetration per 1000 µm^2^ of retina. The size-dependent uptake of the PS beads confirms the trend observed in Figure 5(A). Indeed, the majority of sections originating from explants incubated with 40 nm NPs contain a large amount of beads inside the retina (Figure 6(C), black bar) while the opposite is true for the 100 and 200 nm sized NPs. Figure 6(B) shows representative images of respectively high (>30 NPs/1000 µm^2^ as seen with 40 nm NPs), low (10–30 NPs/1000 µm^2^ as observed with 100 nm NPs) and medium (<10 NPs/1000 µm^2^ for 200 nm NPs). Also on these images, the high VR transfer into the retina of 40 nm beads in comparison with the larger particles is clearly noticeable.

### 3.4. Relevance of vitreous and inner limiting membrane as drug delivery barriers

**Figure 7.**
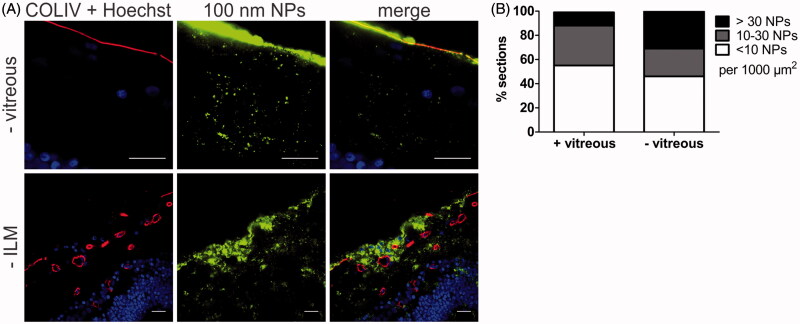
(A) Representative cryosection images showing the transport of 100 nm sized PS beads into the retina, 24 h after applying the beads on the explants. Top row: no vitreous, bottom row: no ILM. ILM and blood vessels (red) are stained by anti-COLIV antibodies, nuclei (blue) with Hoechst, particles are shown in green. Scale bar: 20 µm. (B) Semi-quantitative analysis of 100 nm PS bead uptake in vitreoretinal explants compared to conventional explants without vitreous (*n* = 3).

To illustrate the importance of an *ex vivo* model that keeps the entire VR interface intact, we have compared the transport of 100 nm sized PS beads into the retina using respectively our VR explants and conventional explants without vitreous. As can be derived from Figure 7(B), the transfer of particles into the retina observed using conventional explants was clearly higher. In fact, the fraction of sections containing >30 NPs per 1000 µm^2^ nearly tripled (11–31%). We furthermore observed that absence of vitreous allowed larger particle aggregates to enter the retina (Figure 7(A) – vitreous). In view of these observations, it is evident that tearing off the vitreous significantly affects the transport of NPs into the retina which may result in an inaccurate estimation of the potential of NPs to cross the VR interface. To further explore to which extent the ILM is a transport barrier for NPs, we purposely sought for spots in the retinal sections with a compromised ILM. It has indeed been reported before that the ILM can be severely damaged by tearing of the vitreous (Russell, 2012). A typical image of this case is shown in Figure 7(A) (-ILM) where the ILM is absent in the center of the image, a condition which clearly results in unusually high NP transport.

## 4. Discussion

Intravitreal injection is globally a daily employed administration technique for the delivery of small therapeutics to the retina, such as anti-VEGF medication or antibiotics. For the delivery of gene carriers, on the other hand, subretinal injection is clinically the most investigated technique, especially for targeting photoreceptors or RPE cells in the outer retina. We, and others, however, strongly believe IVT injection can be a worthy alternative for gene delivery to the retina, especially when (widespread) expression over the inner retinal tissue should be obtained (Da Costa et al., 2016; Ochakovski et al., 2017). Yet, to ensure effective gene therapy after IVT injection, gene carriers have to pass the VR interface before reaching the retina. Three parts of this VR interface can be considered as a hurdle: peripheral vitreous, the ILM, and Müller cell endfeet (Figure 1). Strikingly, gene carriers often efficiently overcome these hurdles in small animal *in vivo* studies, usually on rodents, though this success is rarely extrapolated to larger animal models like pigs (Trapani et al., 2015). Based on our knowledge on interspecies differences in ocular anatomy and barrier structure this does not entirely come as a surprise: the combination of the smaller volume of vitreous (5–20 µl) (Lebrun-Julien et al., 2009), its more liquid composition and the reduced ILM thickness make the VR interface in mice hardly representative for the human situation (Skeie & Mahajan, 2013). For this reason, several research groups focus on the culture of large animal retinal explants, however, this usually implies detachment of the vitreous gel so information on the VR interface is lost (Kobuch et al., 2008; Fradot et al., 2011). We therefore aimed to bridge the gap between these *ex vivo* models and the eventual *in vivo* situation in large animals and humans, by developing a novel *ex vivo* explant model based on bovine eyes that maintains the entire VR interface intact. We show that our VR explant remains viable for at least two days and its morphology is rather human-like (Figures 3 and 4). Furthermore, we demonstrate that our model is ideally suited to study the penetration of particles across the VR interface into the retina after IVT injection.

Conveniently, our VR explant model allows for two ways of imaging, where both ways have their merits. The direct imaging method (Figure 2(B)) is faster, less labor-intensive and requires for less tissue manipulation which results in optimal integrity of the whole tissue. The observable tissue depth is, however, limited to the ganglion cell layer. On the other hand, cryosection preparation and imaging (Figure 2(C)) is rather labor-intensive but gives a full cross-section of all retinal layers and allows to stain for a plethora of markers by immunostaining.

We purposely opted for an *ex vivo* approach since this comes with several advantages. Firstly, highly relevant drug delivery studies can be performed on representative large animal species such as pig, cow, or even human donor eyes; this without the high costs that accompany the care and housing of larger species as required for *in vivo* studies. Secondly, *ex vivo* assays are convenient and accessible: eyes can be easily obtained from local slaughterhouses, and only basic cell culture and dissection materials are required to culture our VR model, making it feasible for every researcher. Finally, the application of *ex vivo* assays is in line with the worldwide aim to implement the 3 R principle (replacement, reduction and refinement), originally introduced by Russell and Burch as guidelines for more ethical and less use of laboratory animals (Russell & Burch, 1959).

To demonstrate the value and functionality of our VR explant for drug delivery, we looked into the size-dependent transfer of NPs through the VR interface into the retina. We decided to use commercially available carboxylated polystyrene beads, since these particles are highly fluorescent, well characterized and monodisperse as confirmed by our DLS measurements (Table 1). Furthermore, these particles have potential to penetrate into the retina after IVT injection as: 1) the NP sizes are lower than the estimated mesh size of the vitreal collagen network (550 nm) (Xu et al., 2013) 2) negatively charged particles exhibit ideal vitreal mobility and do not aggregate in the vitreous (Sakurai et al., 2001; Bejjani et al., 2005; Kim et al., 2009; Xu et al., 2013; Martens et al., 2015), and 3) in contrast to positively charged particles, negatively charged ones have been reported before to cross the ILM (Pitkänen et al., 2004; Koo et al., 2012). As anticipated, the PS beads did effectively enter the retina and more importantly, a clear size-dependent trend was noticeable using both imaging methods (Figures 5 and 6): 40 nm PS beads more easily entered the retina from the vitreous than 100 and 200 nm sized beads. It is essential to note that for a given particle size, the number of NPs that crossed the VR interface varied greatly between different retinas and even for different locations within the same retinal explant. For this reason the retinal section data were presented as a distribution rather than only showing the most occurring observation. A potential explanation for this variety in uptake within and between VR explants is that the ILM thickness varies depending on the retinal region (Bu et al., 2015), as well as on the age of the animal (Halfter et al., 2008; Candiello et al., 2010). In addition, although the same particle concentration and injection volumes were injected into the vitreous of the various VR explants, the injection location and the diffusion of particles from the injection spot eventually determine the actual NP concentrations across the vitreous and the retina.

Once NPs passed the VR interface, they were generally located in the inner retinal layers, which is in line with the rationale of exploring IVT injection as a valuable strategy for gene therapy, primarily for targeting cell types in the inner retina. Using the direct imaging method we further observed that 40 nm particles co-localized specifically with Müller cell endfeet (Figure 5(B)), an observation also made by Koo et al. with human serum albumin based nanoparticles in rats (Kim et al., 2009; Koo et al., 2012). Since the Müller cell endfeet abut in the ILM, these cells are likely the first ones particles encounter after IVT injection. Also our *in vitro* uptake studies demonstrate that bovine primary Müller cells efficiently endocytose NPs (data not shown). Unfortunately, we were unable to confirm a clear co-localisation of NPs and Müller cells using cryosection imaging.

Interestingly, while 100 nm sized particles had difficulty crossing the VR interface in our hands, some reports discuss the easy entry of larger particles into the retina (Bourges et al., 2003; Kim et al., 2009; Koo et al., 2012; Apaolaza et al., 2016). Koo et al. for example found 350 nm sized negatively charged human serum albumin particles to easily enter the retina in rats after IVT injection (Koo et al., 2012). Similarly, neutral polylactide particles of the same size were reported to accumulate at the ILM followed by smooth penetration into the rat retina as well (Bourges et al., 2003). Also solid lipid nanoparticles of 230 nm resulted in efficient transfection across nearly all retinal layers after IVT injection in mice (Apaolaza et al., 2016). It should be noted, however, that all these observations were made in rodents, which could account for the contrast with our data obtained in bovine eyes.

To illustrate the importance of preserving the VR interface, we have investigated the uptake of 100 nm sized NPs in conventional explants without vitreous. At the same time, this allows us to explore the specific barrier roles of the different parts of the VR interface. Removing the vitreous had a positive effect on retinal uptake, since the fraction of explants containing high amounts of NPs increased and larger NP aggregates were able to enter the inner layers of the retina (Figure 7(A,B)). Notably, the vitreous does not represent a major barrier for these negatively charged 100 nm PS beads since they are known to have good vitreal mobility (Martens et al., 2013). We therefore expect the effect of vitreous removal to be more pronounced for positively charged particles that are obstructed by their interaction with the negatively charged components of the vitreous (Pitkänen et al., 2003; Peeters et al., 2005; Kim et al., 2009; Koo et al., 2012; Martens et al., 2013; Xu et al., 2013), and for particles that are larger than the mesh size of the vitreal network (∼550 nm) (Xu et al., 2013). Next to losing information on the barrier function of the vitreous for a particular NP, tearing off the vitreous also influences the barrier function of the ILM. In fact, Russell et al. found that vitreous separation commonly results in ILM breaks, ILM evulsion and sporadically even loss of inner retinal layers in cynomolgus monkeys (Russell, 2012). An example of ILM separation upon vitreous removal can be seen in Figure 7(A – ILM), a condition that clearly results in massive NP uptake in the retina. A similar observation was made by Gan et al. who witnessed enhanced penetration of liponanoparticles through the disintegrated ILM of rats suffering from uveitis when compared to healthy rats (Gan et al., 2013). Considering the spectacular contrast in retinal delivery in a retina with and without the ILM, it is obvious that the ILM is a crucial barrier for gene therapy. Seemingly, it functions as a sieve that defines the type and number of particles presented to the inner retinal cells.

The significant role of the vitreous and ILM as a barrier for drug delivery and the influence of vitreous removal on the integrity of both of them again highlights the necessity of the model presented in this study. Certainly, our bovine-derived VR explant could be highly valuable as a relevant set-up to assess if promising particles, showing favorable *in vitro* results, are competent in crossing the different hurdles connected to the VR interface. However, despite its value, some general remarks can be made. It should be noted that by isolating the vitreous along with the retina, the vitreous loses the natural support of the eyecup, causing a partial collapse of the vitreal collagen network (Nickerson et al., 2008). While potential (unwanted) interactions of particles with the vitreal components will still be detected in our model, we refer the reader to other advanced models, such as the one developed by our research group, when exact diffusional rates of fluorescent particles in intact vitreous are to be determined (Martens et al., 2013). Secondly, while the VR explant greatly mimics the *in vivo* situation, certain dynamic processes present in the living eye are not taken into account such as clearance mechanisms and the anterior-posterior flow present in the vitreous (Xu et al., 2000). The currently available *ex vivo* system that most closely resembles the *in vivo* situation, is the perfused eye model. In this approach, an entire eye is isolated, cannulated and perfused, keeping the eye globe perfectly intact (Mains et al., 2011, 2012). This eye integrity is highly beneficial, although it comes with the disadvantage that these perfused eyes are only viable for a limited time (∼9 hours). In contrast, our VR explant remains viable for at least 2 days, making it more suitable to evaluate retinal uptake of carriers after IVT injection. For long-term follow-up of gene carriers, however, *in vivo* studies remain indispensable as for example retinal gene expression usually only reveals itself after 2 weeks (Mowat et al., 2014; Wassmer et al., 2017).

## 5. Conclusions

For future research, we argue that further detailed characterization of the properties and composition of the vitreous and ILM across species could help to identify which models are the most suited for IVT drug delivery studies with the retina as main target. Thanks to the valuable research performed on especially developed large animal models we have the knowledge to smartly design vectors able of overcoming the vitreous as a barrier. In contrast, owing to the diversity of vectors studied on a variety of species the physicochemical requirements to efficiently cross the ILM is way less coherent. Well-designed systematic studies into which particle properties do result in successful retinal entry from the vitreous could therefore form a sound foundation for future design of ‘the’ optimal gene carrier administered by IVT injection. We are strongly convinced that advanced *ex vivo* models, such as the one presented in this paper, could have great significance in reaching this goal. Surely, our VR explant is currently the most representative *ex vivo* model on the market that is viable for a sufficiently long time to study carrier uptake. Moreover, our *ex vivo* approach is readily accessible, relatively cheap and transferrable to other large species like pig or human.
